# Current HRD assays in ovarian cancer: differences, pitfalls, limitations, and novel approaches

**DOI:** 10.3389/fonc.2024.1405361

**Published:** 2024-08-16

**Authors:** Federica Guffanti, Ilaria Mengoli, Giovanna Damia

**Affiliations:** Laboratory of Preclinical Gynaecological Oncology, Department of Experimental Oncology, Istituto di Ricerche Farmacologiche Mario Negri IRCCS, Milan, Italy

**Keywords:** homologous recombination deficiency, ovarian cancer, predictive biomarkers, PARP inhibitors, platinum drug, artificial intelligence

## Abstract

Ovarian carcinoma (OC) still represents an insidious and fatal malignancy, and few significant results have been obtained in the last two decades to improve patient survival. Novel targeted therapies such as poly (ADP-ribose) polymerase inhibitors (PARPi) have been successfully introduced in the clinical management of OC, but not all patients will benefit, and drug resistance almost inevitably occurs. The identification of patients who are likely to respond to PARPi-based therapies relies on homologous recombination deficiency (HRD) tests, as this condition is associated with response to these treatments. This review summarizes the genomic and functional HRD assays currently used in clinical practice and those under evaluation, the clinical implications of HRD testing in OC, and their current pitfalls and limitations. Special emphasis will be placed on the functional HRD assays under development and the use of machine learning and artificial intelligence technologies as novel strategies to overcome the current limitations of HRD tests for a better-personalized treatment to improve patient outcomes.

## 1 Introduction

Ovarian cancer is a heterogeneous disease, including different subtypes. Depending on the cells of origin, it can be classified into ovarian carcinomas (OC), sex cord-stromal tumors, or germ cell tumors (1, 2). OC is the most common type, accounting for 90% of tumors, and five different histological subtypes can be recognized (high-grade serous (HGSOC), low-grade serous, clear cell, and mucinous) with different molecular features, clinical characteristics, and response to therapy (2). The most prevalent histotype (75% of the cases) is the HGSOC (2), which still has a poor prognosis due to the lack of effective screening tests preventing its early diagnosis; in addition, even if it is very responsive to a platinum-based therapy, most of the patients will eventually relapse with a much less chemosensitive tumor (3, 4).

In the last decades, the molecular studies behind HGSOC have outlined that 50% of these tumors have defects in homologous recombination (HR) repair (5). HR represents a relatively error-free pathway that repairs the DNA double-strand breaks (DNA-DSBs), the most cytotoxic cellular lesions when cells are in the S/G2 phases of the cell cycle where sister chromatids are available, while the non-homologous end joining (NHEJ) pathway occurs along all the cell cycle to fix DSBs in a more rapid, even if low-fidelity manner (6). The functional inactivation of HR repair, a condition known as *BRCAness* or HR deficiency (HRD), is due to mutations and/or epigenetic modification (i.e., promoter hypermethylation) in genes involved in the HR pathway, including *BRCA1*, *BRCA2*, *ATM*, *BARD1*, *MRE11*, *RAD51*, *PALB2*, *Fanconi Anemia* genes, and many others [for an exhaustive review, please refer to (7)]. While a tumor with a deficiency in HR has the probability to accumulate much more DNA damage than a HR-proficient (HRP) tumor, this characteristic can also be considered its “Achilles heel” as it renders it very susceptible to the cytotoxic effects of platinum-based drugs and to poly (ADP-ribose) polymerase inhibitors (PARPi). Platinum drugs require HR for the repair of the intrastrand cytotoxic lesions they induce in the DNA, so cells with inactivation of HR are extremely sensitive to cisplatin (8, 9).

As regards PARPi, synthetic interaction has been shown to be the basis of their activity on HRD cells [for a recent updated review, please refer to (10)] and has revolutionized the therapeutic approach in the last decade in OC.

In OC, the *BRCAness* or HRD phenotype identifies a subgroup of tumors that are generally HGSOC, display high response rates to first and subsequent lines of platinum therapy, have long progression-free intervals between recurrences, and have a better overall survival (OS) (8, 11). Recently, it has also become an eligible criterion for treatment with PARPi, and its testing has been strongly recommended by both the National Comprehensive Cancer Network (NCCN) (12) and the European Society for Medical Oncology (ESMO) guidelines (13).

We will here review the different types of genetic HRD tests available, with a particular focus on the new academic ones, on the functional HRD tests, and how the emerging role of artificial intelligence (AI) can help in a better and more specific definition of HRD in OC.

## 2 Clinically approved HRD tests: description, applications, and limitations

The functional inactivation of HR can be determined either by looking for germline or somatic mutations in *BRCA1/2* genes (g*BRCA1/2* and s*BRCA1/2*), as BRCA1/2 proteins play a pivotal role in HR repair pathway and their loss causes a defective DNA-DSB repair, or by assessing the presence of genomic alterations deriving from unproperly repaired DSBs by low-fidelity repair mechanisms like NHEJ or microhomology-mediated end joining (MMEJ) (14). With the elucidation of the different genes involved in HR, the mutational status of other genes beyond *BRCA1/2* has also been included when defining HRD (15). The genomic alterations deriving from a defective/inefficient HR include insertions and deletions, copy number alterations, and structural chromosomal rearrangements. Their combination in specific patterns is detected as a loss of heterozygosity (LOH), telomeric allelic imbalance (TAI), and large-scale state transitions (LSTs), generally referred to as “genomic scars”, since they remain as footprints in the genome of a cell lacking HR (16). Specifically, LOH represents the loss of one parent’s allele contribution at a specific locus, either by deletion or deletion and simultaneous duplication of the remaining mutated allele generating homozygosity (17). Abkevich et al. investigated the association between HR defects and genomic patterns of LOH in two cohorts of epithelial ovarian tumors, finding a highly significant correlation between a HRD score defined as the number of LOH regions of intermediate size (> 15 Mb, but shorter than the length of the whole chromosome) and HR deficiency, measured by *BRCA1/2* and *RAD51C* mutations (18). TAI refers to an allelic imbalance extending from the break-point to the subtelomeric region without including the centromere. It was found to predict sensitivity to platinum treatment in HGSOC patients with wild-type (wt) *BRCA1/2* and proposed as a marker of impaired DSB repair (19). LSTs are defined as chromosomal breaks between two adjacent regions of at least 10 Mb in size by Popova et al. (20), who also associated this genomic signature with *BRCA1/2* inactivation in two independent series of basal-like breast carcinomas.

A strong association of these three independent genomic signatures with *BRCA1/2* deficiency was found in 215 samples of breast tumors, regardless of the cancer subtype, demonstrating that they correlate with each other and that they all measure the same genomic alteration (21). A combined HRD score, calculated as an unweighted sum of the LOH, TAI, and LST scores, was also produced and turned out to be a much more robust predictor of HRD than the individual scores (21), and its predictive role was assessed and validated in three neoadjuvant triple-negative breast cancer (TNBC) trials (22).

The evaluation of these genomic alterations has been the basis for the two currently Food and Drug Administration (FDA)-approved genomic HRD tests that have been used as companion tests in the clinical development of PARPi (23): the Myriad MyChoice CDx and the FoundationOne CDx. The Myriad MyChoice CDx (Myriad Genetics, Salt Lake City, UT, USA) assay takes into account both the presence of mutations and rearrangements in *BRCA1/2* genes and a Genome Instability Score (GIS), which is calculated by combining LOH, TAI, and LST measurements deriving from single-nucleotide polymorphism (SNP)-panel sequencing. The threshold > 42 was set to distinguish HRD-positive and HRD-negative tumors (24, 25). The FoundationOne CDx (Foundation Medicine, Boston, MS, USA) test relies on the calculation of the percentage of LOH (%LOH) with a cut-off of > 16% to discriminate between LOH-high and LOH-low tumors and on the sequencing of an extensive panel of genes, including *BRCA1/2* and HR-related genes (26, 27).

Both Myriad MyChoice and FoundationOne tests have been implemented in phase III clinical trials testing PARPi in OC and have demonstrated an association with the response to therapy for PARPi (28). The PRIMA trial evaluated niraparib maintenance vs. placebo in patients with newly diagnosed advanced OC after response to platinum-based chemotherapy (29). The Myriad MyChoice test clustered patients in HRD-negative and HRD-positive groups, with the latter having *BRCA1/2* alterations and/or a GIS ≥ 42 (22). The niraparib effect vs. placebo was evaluated as progression-free survival (PFS) in the overall population (13.8 months vs. 8.2 months), in the HRD-positive subgroup with *BRCA1/2* mutations (21.9 months vs. 10.4 months), in HRD-positive patients without *BRCA1/2* mutations (22.1 months vs. 10.9 months), and in patients without HRD (8.1 months vs. 5.4 months). All niraparib-treated subgroups showed improvement of PFS, much more evident in HRD patients; however, in HRP patients, niraparib treatment was effective, even if less than in patients with defective HR, and regardless of *BRCA1/2* mutational status (29). These results led to niraparib approval by the FDA for advanced OC in the maintenance setting after tumor response to first-line chemotherapy, regardless of *BRCA1/2* mutational status and HR status. The PAOLA-1/ENGOT-ov25 phase III trial aimed at evaluating the addition of olaparib to bevacizumab vs. olaparib plus placebo in maintenance following chemotherapy and bevacizumab cotreatment in newly diagnosed advanced OC (30). Again, based on the Myriad MyChoice CDx cut-off of ≥ 42, patients were classified as having HRD or HRP; the presence of *BRCA1/2* mutations was also taken into account. Olaparib-treated patients showed an increased median PFS compared to placebo-treated patients in the overall population (22.1 vs. 16.6 months), in *BRCA1/2*-mutated patients (37.2 months vs. 21.7 months), in HRD-positive tumors having GIS ≥ 42 and/or *BRCA1/2* alterations (37.2 vs. 17.7 months), and in HRD-positive tumors without *BRCA1/2* mutations (28.1 months vs. 16.6 months), leading to the FDA approval of the combination of olaparib and bevacizumab in the maintenance setting in HRD-positive advanced OC beyond *BRCA1/2* mutations (30). The VELIA trial assessed the addition of veliparib to carboplatin/paclitaxel chemotherapy (veliparib combination only group) compared to veliparib addition both during chemotherapy and in the maintenance setting (veliparib-throughout groups) in OC patients (31). In this study, the threshold for determining HRD with the Myriad test was lowered to 33 to try to improve the sensitivity of the test for the identification of PARPi responders. However, no difference between the control group (chemotherapy only) and the veliparib-throughout approach could be appreciated in terms of PFS in the *BRCA1/2* wt subgroup between HRD-positive and HRD-negative patients (31) in contrast with the previously cited results (29, 30).

The FoundationOne CDx test was adopted in the phase III ATHENA-MONO trial, designed to evaluate rucaparib activity in first-line maintenance after response to chemotherapy in advanced HGSOC (32). Patients were stratified based on FoundationOne CDx test results and *BRCA1/2* status into wt*BRCA1/2*/LOH-high (LOH ≥ 16%; HRD-positive), w*tBRCA1/2*/LOH-low (LOH < 16%; HRD-negative), and wt*BRCA1/2*/LOH undetermined (32). Rucaparib maintenance treatment significantly improved PFS vs. placebo in patients regardless of *BRCA1/2* mutations and HRD status, as the median PFS in the HRD population was 28.7 months vs. 11.3 months, 20.2 months vs. 9.2 months in the intention-to-treat population, and 12.1 months vs. 9.1 months in the HRD-negative population.

The implementation of these genomic HRD tests in phase III clinical trials allowed to broaden the patient population benefiting from PARPi in the first-line maintenance setting, as compared to their initial use in women with newly diagnosed advanced OC and germline or somatic *BRCA1/2* mutations (33, 34).

PARPi antitumor activity has also been investigated for recurrent advanced OC, and in particular as maintenance therapy in platinum-sensitive relapsed OC patients in Study 19 (35–37) and SOLO2 (38, 39) trials (olaparib), NOVA trial (40) (niraparib), and ARIEL3 study (26, 27) (rucaparib). Based on Study 19 (35–37) and SOLO2 (38, 39) results, where no HRD genomic tests were applied, the FDA approved olaparib for this condition regardless of *BRCA1/2* mutational status, as both mutated and wt tumors responded to the treatment. In the NOVA phase III clinical trial (40), a g*BRCA*-mutated cohort and a non-g*BRCA* cohort with platinum-sensitive relapsed cancer were randomized to niraparib or placebo. All the patients also underwent HRD testing with Myriad MyChoice CDx, and the GIS cut-off of ≥ 42 was used to define HRD-positive tumors. PFS with niraparib compared to placebo was 21 months vs. 5.5 months in the g*BRCA* cohort, 9.3 months vs. 3.9 months in the non-g*BRCA* cohort, and 12.9 months vs. 3.8 months in the non-g*BRCA* cohort with a HRD score ≥ 42 (inclusive of patients with somatic *BRCA1/2* mutations). An exploratory analysis on subgroups of the non-g*BRCA* cohort showed an increase in PFS in tumors with the s*BRCA* mutation (20.9 months vs. 11 months, niraparib-treated vs. placebo, respectively), HRD-positive/wt*BRCA1/2* tumors (9.3 months vs. 3.7 months), and HRD-negative/wt*BRCA1/2* tumors (6.9 months vs. 3.8 months) (40). Since the treatment resulted in effectiveness regardless of the presence or absence of g*BRCA1/2* mutations or HRD status, niraparib was approved for the maintenance therapy of relapsed advanced OC in patients previously treated with platinum chemotherapy and still responsive to it regardless of specific biomarkers (40). However, more recently, the FDA restricted niraparib indication only to patients with g*BRCA1/2* mutations, as in terms of median OS, there was no difference between niraparib and placebo non-g*BRCA1*-mutated patients (41).

The ARIEL3 phase III study was designed to evaluate the rucaparib effect in maintenance after response to second-line or later platinum-based chemotherapy, and the FoundationOne CDx HRD test was used to identify HRD OC patients with a threshold of ≥ 16% to define HRD cases. In ARIEL3, improvement of PFS of rucaparib vs. placebo was observed in all subgroups of patients: in *BRCA1/2*-mutated (deleterious germline or somatic) (16.6 months vs. 5.4 months), in patients with HRD tumor (defined as g/s*BRCA1/2*-mutated or wt*BRCA1/2* with high LOH) (13.6 months vs. 5.4 months), and in the intention-to-treat population (10.8 months vs. 5.4 months) (26). Within the *BRCA1/2* wt cohort, both LOH-high (%LOH ≥ 16%) and LOH-low (%LOH < 16%) groups benefitted from rucaparib compared to placebo, although to a lesser extent than in the LOH-low subgroup (9.7 months vs. 5.4 months for wt*BRCA1/2*/LOH-high; 6.7 months vs. 5.4 months for wt*BRCA1/2*/LOH-low) (26). Given the effects observed across all the groups, rucaparib was FDA-approved for maintenance therapy in platinum-sensitive recurrent OC, regardless of *BRCA1/2* mutations or HRD status (26, 27).

Two recent phase II clinical trials evaluating PARPi in the recurrent setting also used these HRD tests to assess PARPi efficacy. The LIGHT study investigated olaparib treatment in patients with platinum-sensitive relapsed ovarian cancer (who received at least one prior cycle of chemotherapy). In total, 272 patients were enrolled, and 259 were assigned, according to the presence of *BRCA1/2* mutations and the HRD status determined by the Myriad MyChoice assay, to four predefined cohorts: g*BRCA1/2*-mutated patients, s*BRCA1/2*-mutated patients, HRD-positive (GIS ≥ 42) with no *BRCA1/2* alteration patients, and HRD-negative (GIS < 42) patients. Response to olaparib was observed in all the cohorts, with BRCA-mutated patients showing the highest overall response rates (ORR) (69% and 64% in the gBRCAm and sBRCAm cohorts, respectively) and longest PFS (~ 11 months) (42). The QUADRA phase II clinical trial was conducted to investigate the role of niraparib in patients with recurrent OC after three or more lines of therapy being sensitive, resistant, or refractory to platinum. The Myriad MyChoice assay was used to discriminate HRD-positive and HRD-negative tumors based on the cut-off of ≥ 42. ORR and clinical benefits of niraparib were greater in platinum-sensitive patients compared to the resistant and refractory subgroups. Across all platinum subgroups, the ORR were 29% for g-s*BRCA1/2*-mutated, 15% for HRD-positive tumors (including *BRCA1/2*-mut and wt), and 3% for HRD-negative (wt*BRCA1/2* and test score < 42) or unknown. Based on these data, niraparib was FDA approved for patients treated with at least three chemotherapy regimens whose cancer either harbored a deleterious *BRCA1/2* mutation or was platinum-sensitive with evidence of HRD (43).

Myriad MyChoice and FoundationOne assays rely on different molecular bases, and they are not equivalent. Indeed, Timms et al. evaluated the correlation and the positive percentage agreement (PPA) among the Myriad MyChoice (threshold scores of 42 and 33 were considered), the %LOH, and the presence of a pathogenic mutation in an 11-HR gene panel (44). Whole-genome SNP analysis was used to calculate the MyChoice HRD score and %LOH in two cohorts of 3,278 and 248 patients. The mutations in 11 genes of the HR pathway (i.e., *ATM*, *BARD1*, *BRCA1*, *BRCA2*, *BRIP1*, *CHEK2*, *MRE11A*, *NBN*, *PALB2*, *RAD51C*, and *RAD51D*) were also evaluated for a subset of tumors from the second cohort. HRD-positive tumors were defined based on either 42 or 33 threshold scores for the Myriad test, 16% cut-off for %LOH, as well as the presence of a pathogenic variant in one of the 11 studied HR genes. The correlation between positive results from %LOH and the 11-gene panel was compared to the Myriad HRD test. Overall, 19%–61% of patients were identified as positive by Myriad HRD and would have been missed by %LOH or by the 11-gene panel in these two cohorts, suggesting these HRD tests are not equivalent and should not be considered interchangeable in predicting PARPi response. Nevertheless, it is evident how the application of HRD genomic tests allowed the tailored use of PARPi in OC, improving HGSOC patients’ prognosis (45).

The implementation of the Myriad MyChoice CDx and the FoundationOne CDx in the clinic is favored by the fact that SNP-sequencing and mutational analysis of *BRCA1/2* and HR-related genes are performed on DNA samples extracted from FFPE tumor tissues, generally available after primary debulking surgery or from tumor biopsies at diagnosis (14). However, their application has some limitations, especially with regard to the preanalytical phase: insufficient material due to previous cycles of neoadjuvant therapy, paucity of tumoral cells, or poor quality of the specimen are all factors strongly affecting the success rate of the HRD tests (46). Also, fixation artifacts can compromise the quality of the tissue, and so the quality of the extracted DNA and HRD test result, possibly generating false-positive, false-negative, or inconclusive results occurring in 10%–15% of the cases (47). In addition, because of the intratumor heterogeneity, the same tumor could be defined as HRD-positive or HRD-negative depending on the biopsy site (14). Moreover, both Myriad MyChoice and FoundationOne CDxs are performed in a centralized way, so the samples have to be sent abroad to be analyzed, with no control over the parameters during the analytical step and a with turnaround time of 18 days for the MyChoice test (14).

Recent work from Denkert et al. (48) evaluated the transfer of the Myriad MyChoice assay in an academic molecular pathology laboratory through the parallel assessment of 514 OC samples in both Myriad’s laboratory and a decentralized one. 498 samples out of 514 provided an identical GIS status, determined with a sensitivity of 94.6% and a specificity of 98.4%, resulting in a GIS-status concordance of 96.9% between the two laboratories. Similarly, the feasibility of HRD testing implementation in a decentralized pathological department was demonstrated by Heitz et al. (49), getting test results in 514 HGSOC with a 96.9% (*p* < 0.00001) concordance between Myriad and academic laboratories for the GIS status, with a sensitivity of 94.6% and a specificity of 98.4%. In addition, the concordance for HRD status was even higher at 97.1% (499 of 514 tumors) (49).

## 3 An overview of novel academic genomic HRD tests (technical aspects, advantages, and caveats)

The European Society of Gynecological Oncology (ESGO) and the European Society for Medical Oncology (ESMO) have recently recommended testing all patients diagnosed with nonmucinous ovarian, tubal, and peritoneal cancers for *BRCA1/2* mutations, suggesting the two commercially available and clinically approved HRD genomic tests as good predictive tools for PARPi benefits and pointing out insufficient knowledge regarding no-BRCA/HRD-related mutational signature-based predictive biomarkers (50, 51). The decentered implementation of HRD tests would have the advantage of reducing costs and logistic problems (extra-time waiting to send samples and to receive the results from accredited centralized laboratories); in addition, the development of high-quality genomic assays based on the most advanced sequencing technologies would provide accurate results on HR-related mutational signatures and could eventually lead to harmonized results (23, 30, 52). Trying to overcome the current limitations and with the goal of having robust, feasible, closer to patients, less expensive, and more sensitive HR tests, several research laboratories have been actively involved in setting up new in-house HR tests. The European Network of Gynecological Oncology Trial (ENGOT) HRD Initiative (EHEI) is a large collaboration of European academic research centers to find an academic alternative to commercially available HRD assays and novel, reliable HRD biomarkers (53) (**Table 1**). The EHEI aims to correlate the performance of different academic genomic and functional HRD assays with the Myriad MyChoice test, evaluating the tumor samples of patients enrolled in the phase III trial. Seven out of 20 research laboratories satisfied the inclusion criteria (tests mainly based on non-BRCA/HR gene mutation panels or did not reach capability, financial, or regulatory standards were excluded), and data published until now seem encouraging (53).

**Table 1:** Overview of current academic DNA-based HRD tests under investigation.

Research group	HRD test name	Affiliation	Concept and underlying technologies	Reference
Mango IT	n/a	Humanitas University, Milan, Italy	WES-scoring algorithm based on the presence of LOH	(54)
SAKK	Geneva test	Hôpitaux Universitaires de Genève, Switzerland	HRD phenotype score based on whole-genome CNV (ThermoFisher Oncoscan SNP assay, Waltham, MS, USA)	(55)
BGOG	Leuven HRD test	University Hospitals Leuven, Belgium	Capture-based targeted NGS SNP panel	(56)
GINECO	GIInger	Centre Léon BERARD, and University Claude Bernard Lyon I, Lyon, France	HRD solution developed with SOPHiA GENETICS. Targeted sequencing (28 genes) low coverage WGS. Proprietary deep learning algorithm	(57)
AGO	BRCA-like Copy-Number Aberration Classifiers	Netherlands Cancer Institute, Amsterdam, the Netherlands; University Hospital Cologne, Cologne, Germany	BRCA-like copy number profile based on low coverage WGS	(58)
NOGGO	NOGGO	Institut für Hämatopathologie Hamburg, Germany; Charité-Universitätsmedizin Berlin, Germany	LOH scarring	(59)
–	ShallowHRDv2	Institut Curie and Paris Sciences Lettres Research University, Paris, France	Software tool trained on shallow WGS data	(60)
Other academic HRD tests				
**Research group**	**HRD test name**	**Affiliation**	**Concept and underlying technologies**	**Reference**
Department of Medical Genetics; Early Cancer Institute	HRDetect	University of Cambridge, Cambridge UK	WGS mutational signature-based HRD classifier	(61–63)
–	GIScar	Centre François Baclesse, Caen, France	Genomic Instability Score	(64)
Department of Laboratory Medicine and Pathology	LOH score	University of Washington School of Medicine, Seattle, WA	Targeted, Hybridization capture and NGS of genome-wide SNP sites to calculate LOH	(65)

In a recent study, two academic genomic tests and a functional assay were compared to the Myriad test in a retrospective cohort of 100 untreated, randomly selected HGSOC patients participating in the MITO16A/MaNGO-OV2 trial (54). These two NGS-based academic methods evaluated genomic instability. Both tests showed good concordance with Myriad MyChoice in terms of HRD assessment and correlation with patients’ prognosis and platinum therapy outcome in a multivariate analysis (54). The functional test, evaluating the basal level of RAD51 foci, did not perform well as only a small sample size could be evaluated and the concordance rate with Myriad was low (37%). These results were corroborated by Scaglione et al., who compared their shallow whole-genome sequencing HRD test (named *HRD-MITO* assay) to another shallow NGS method and to the gold standard Myriad MyChoice test in 20 retrospective HGSOC chemotherapy-naïve clinical samples (66).

Other academic groups within the EHEI developed DNA-based HRD tests, relying on different technologies (**Table 1**). The *Leuven HRD* test is based on a capture-based targeted NGS SNP panel and has been shown to significantly correlate with Myriad MyChoice CDx PLUS, and similar differences in PFS and OS were observed between HRD-positive and HRD-negative populations as the reference test in 468 advanced OC samples from the PAOLA-1/ENGOT-ov25 trial (56). The whole-genome CNV assay (*Geneva HRD* test) is based on OncoScan plus a normalized large-scale transition (nLST) test (55); the *NOGGO* test involves the LOH scarring (*NOGGO* test) (59); low coverage Whole Genome Sequencing (WGS) for BRCA-like copy number profile (BRCA-like Copy-Number Aberration Classifiers) (58, 67), and low coverage WGS associated with a deep learning algorithm (*GIInger*) (57). Very recently, the shallowHRDv2 test was developed, based on the work of Eeckhoutte et al., who were the first to describe a software tool named *shallowHRD* to detect HRD based on WGS at low coverage (1×) (68). This test was validated in the 449 FFPE samples of the PAOLA-1/ENGOT-ov25 patients (60). The ShallowHRDv2 test and Myriad MyChoice had substantial overall agreement, but the first had a lower failure rate (3% vs. 11%) and could also predict the PARPi benefit in terms of OS in patients treated with olaparib and bevacizumab compared to bevacizumab and placebo treatment. In addition, the test had positive results for those patients who had inconclusive results with the MyChoice test (60). Compared with other high-throughput WGS-based approaches evaluating HRD, such as HRDetect (WGS 10×) (61, 62), shallow WGS HRD tool is linear, time-saving, and low-cost.

Even if all these academic solutions are based on different advanced sequencing technologies, they were similar or even better than Myriad MyChoice, with lower failure rates, similar or higher sensitivity, and a prognostic role in the retrospective analysis of PAOLA-1/ENGOT-ov25 patients (54–57, 59, 60). These findings support their clinical use since most of them can be easily integrated into the laboratory routine, representing less expensive alternatives to commercial tests. However, while testing the different academic HRD biomarkers on an identical set of trial samples enabled the elucidation of the strengths and limitations of each assay, prospective validation studies are mandatory.

Outside the EHEI, other noteworthy in-house HRD tests have been developed. The GIScar is an academic genomic instability score developed through targeted sequencing of a 127-gene panel to determine HRD status and has been compared with the standard Myriad test in a subgroup of 469 DNA tumor samples from the PAOLA-1/ENGOT-ov25 trial, and its predictive value for olaparib maintenance therapy plus bevacizumab was evaluated (64). GIScar demonstrated good feasibility and optimal concordance with the reference test to classify HRD samples, with a lower rate of undetermined results (1% vs. 9%). Both PFS and OS hazard ratios with GIScar were similar to the standard test, but interestingly, tumors identified as HRD by GIScar and determined as HRP by Myriad had better PFS with olaparib (HR, 0.23; 95% CI, 0.07–0.72), indicating a higher ability to identify patients who may benefit from olaparib maintenance therapy (64).

The HRDetect score is based on a DNA mutational signature-based algorithm and is a weighted model of six mutational signatures associated with HRD, including microhomology-mediated deletions, base substitutions, and rearrangement signatures besides genomic scars (61). The HRDetect test showed good performance in predicting BRCAness phenotypes in breast, ovarian, and pancreatic cancer, identifying BRCA-deficient tumors in FFPE samples with a narrow edge of error (61). Interestingly, this assay also considers epigenetic alterations in the *BRCA1* promoter, and it has been able to identify a HRD phenotype in one-third of *BRCA* wt tumors without known HR repair gene mutations (61). The HRDetect assay has been tested in 43 triple-negative breast cancer patients in the phase II RIO trial aimed at investigating the activity of PARPis and has been shown to be more specific to detecting HRD patients than a LOH/copy number-based HRD score (62). In a cohort of OC samples, HRDetect displayed a sensitivity of 100% in identifying BRCAness patients, as opposed to 60% of the GIS (22). More recently, the HRDetect assay has also been used to characterize OC patient-derived xenograft (PDX) models, and the HRD status is correlated with low RAD51 foci expression at the basal level in the PDXs (69). Mutational signature analysis and HRDetect score identified a subgroup of ATRi responders in a panel of 112 metastatic colorectal cancer preclinical models, suggesting its feasibility in different cancers and its potential as an HR biomarker (63).

Even these latter tests have some limitations, including the need to set up and validate thresholds that may vary among different cancer types, differences among the SNP panels, and the analytical pipeline adopted. For example, Krumm et al. LOH score cut-off of ≥ 11% is lower than ≥ 16% of the FoundationFocus CDX assay (70), which may be due to the different set of target SNPs used or differences in bioinformatic pipelines.

There are, however, some more common technical issues involving all the described tests: the need for minimum tumor cellularity in the samples to be analyzed, which could be hard to get in small bioptic samples; and the need to optimize and standardize sample processing and preanalytical analyses (52). In addition, the tumor heterogeneity, which could partially explain the differences observed between experimental tests and the reference ones, needs to be taken into account, and test validation on samples taken from different metastatic sites of the same patient should also be carried out. Nevertheless, the main limitation of these genomic assays is the inability to give a read-out of the effective HR functionality and to capture tumor evolution events. Indeed, the genomic scars imprinted by the lack of a functional HR are not removed by the subsequent restoration of HR (by a reversion mutation in HR genes) that is associated with PARPi/platinum resistance, hampering their ability to predict the development of resistance.

## 4 Functional methods to assess HR repair status

**Figure 1:**
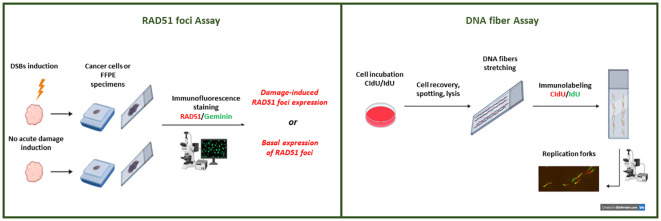
Representation of the HRD functional tests under development. A schematic view of the immunofluorescent-based RAD51 foci tests. RAD51 foci can be quantified after their formation following DNA-DSB induction (through radiation or pharmacological treatment) or at the basal level (without DSB induction). Only proliferating (i.e., geminin positive) cancer cells in specimens are considered to evaluate nuclear RAD51 foci expression. Overview of the DNA fiber assay technique. Nascent DNA is labeled with nucleotide analogs (CldU and IdU) in cancer cells (2D or 3D *in vitro* cultures). Cell lysate solution for DNA spreading is spotted onto microscope slides and allowed to run the length of the slide. After spreading and fixation, the DNA fibers are immunostained and images are acquired for analysis.

HR functional assays represent a promising approach aiming at identifying HRD tumors and providing a readout of the tumor’s HR status in real time, independently from the upstream events leading to HR dysfunction. These characteristics make functional tests extremely appealing, especially in the clinical setting of acquired resistance to DNA-damaging agents and PARPi. In fact, genomic assays failed to identify an eventual HR-restored activity when genetic reversions or secondary mutations occur in *BRCA1*, *BRCA2*, or other HR genes, representing the most common clinical mechanism of acquired resistance to PARPi (71, 72). We will discuss two types of functional tests (**Figure 1**).

### 4.1 RAD51 foci tests

RAD51 is a nuclear recombinase playing a central role in HR and in replication fork processing (73, 74). RAD51 is able to aggregate and form long nucleoprotein filaments mainly driven by BRCA2 on single-stranded DNA 3′ overhangs when a DSB occurs, which can be visualized as local spots of protein accumulation when DSBs occur, named foci. RAD51 foci are microscopically detectable after immunofluorescence (IF) or immunohistochemistry staining, and their formation is a downstream event in HR and occurs when the sister chromatid is available, leading to strand invasion and the homologous sequence of the intact sister chromatid being used to repair DSBs in an error-free way (15, 75). Since the inability to form RAD51 foci is a common feature of HRD, their detection is a suitable functional biomarker of HRD (76). RAD51 foci formation was first investigated in 2009 by Willers et al. in fresh *ex vivo* biopsies from advanced breast cancer patients after ionizing radiation (IR) treatment, and they reported that lower RAD51 foci formation induction was associated with a *BRCA1*-deficient phenotype (77). However, Mukhopadhyay et al. were the first to analyze RAD51 foci induction levels by IF assay in primary cultures from ascitic fluid in OC patients and demonstrated that < 2-fold rise of RAD51 foci formation after induction of DSBs in cancer cells correlated with functional HRD status of the tumors, greater responsiveness to PARPi and platinum, as well as a higher median survival in 50% of OC patients (78, 79). Greaser et al. described the RAD51 foci detection in *ex vivo* breast cancer organotypic biopsies collected one day after treatment with anthracycline-based chemotherapy. Notably, in this study, RAD51 foci were quantified by IF, considering cells positive to both RAD51 foci and geminin, a marker of G2/S cell cycle phases, when the HR is specifically activated (80, 81). The RAD51 score was defined as the percentage of cells geminin-positive and expressing at least one RAD51 focus per nucleus (82). A RAD51 score < 10% was observed in 26% of the cases, and this subgroup was significantly enriched in Triple Negative Breast Cancer (TNBC) harboring HR defects and strongly predicted response to chemotherapy (82). The analysis of RAD51 foci expression in cancer samples after IR damage establishes the path for the standardization of the REcombination CAPacity (RECAP) test (83–88). The RECAP functional test mainly relies on RAD51 foci formation quantification after IR on fresh tumor tissues within 2 h of treatment. It was initially used on fresh primary breast cancer samples whose BRCA status was known, and a strong correlation between RECAP score and BRCA defects was found. Of the 23 RECAP HRD tumors, 16 were *BRCA1/2* mut or had *BRCA1* promoter hypermethylation or *BRCA*-associated large genomic rearrangements, while seven scored HRD were non-BRCA related (84). When the RECAP test was used on 44 metastatic breast cancer *ex vivo* biopsies, the RECAP HRD tumors were 13, and five harbored germline *BRCA* mutations. The RECAP test also identified three tumors as HRP, correlating with post-treatment HR restoration after *in vivo* progressive disease on platinum and PARPi treatment, which was explained in one patient by a secondary *BRCA1* revertant mutation (89). Meijer et al. tested the RECAP method in parallel with two DNA-based tests to identify HRD cases in a cohort of primary and metastatic breast cancer (85). Even though only 70% of concordance was reported for both RECAP-Classifier of Homologous Recombination Deficiency (CHORD) test and the RECAP-BRCA1/2-like classifier, RECAP identified HRD tumors that DNA-based tests did not capture, suggesting the need to compare these HRD tests in clinical trials (85). The RECAP test was also used in HGSOC and endometrial cancer samples (86, 90), and in these settings, the sensitivity of the test in identifying HRD tumors was 100%, with no *BRCAness* phenotype observed in the RECAP-HRP-defined samples (84, 86, 90). Its prognostic potential has been assessed in two studies, where a trend toward better OS in HGSOC patients with low RECAP scores treated with platinum-based therapy has been reported (86, 91). Tumiati et al. reported that specimens collected from omentum, ovary, or metastases of the same ovarian cancer patient have different RAD51 scores, supporting the idea that drug-resistance derives from the selection of subclones and enforcing the role of intratumor heterogeneity as a possible cause of platinum resistance (92). However, despite the potential of RECAP as a HRD functional test, it is limited by the fact that it needs IR treatment of fresh tumor tissues, hampering its use in clinical practice.

To overcome these limitations, two seminal articles in 2018 reported that RAD51 foci expression could be quantified in FFPE tumor samples at the basal level using a simple IF technique (93, 94) (**Figure 1**). Cruz et al. set up a method to quantify RAD51 foci in proliferative/geminin-positive cells in untreated breast cancer FFPE samples and determined a RAD51 foci score able to discriminate PARPi-sensitive (low score) from resistant (high score) tumors (93). Specifically, a cut-off of ≤ 10% of RAD51/geminin-positive cancer cells was evaluated in different areas of the FFPE tissue, associated with a HRD phenotype (86). Similarly, Castroviejo-Bermejo et al. demonstrated that a low basal expression of RAD51 foci in geminin-positive breast cancer cells correlated with olaparib sensitivity in *BRCA* wt tumors, thus identifying potential HRD/*BRCA* wt cases (94). The RAD51 foci/FFPE test was validated in PDX models of OC, breast, and prostate cancer, where a low RAD51 foci score (≤ 10%) was associated with the PDXs’ HRD status response to PARPi, while association for platinum-based therapy was contrasting (69, 95). While preclinical works validated RAD51 foci as a robust HRD biomarker in breast and HGSOC clinical samples (87, 88, 96–99), when compared with approved Myriad MyChoice or other academic genomic tests in HGSOC patients enrolled in the MITO16A/MaNGO-OV2 trial, its performance was low, with a failure rate of 30% with respect to the 2% of the DNA-based assays (54). These suboptimal results were explained by the quality of the FFPE samples of ovarian surgical specimens (54), requiring further validation and calibration of the method. Kramer et al. assessed the validity and reproducibility of the IF-based RAD51 test in four different academic laboratories on 22 HGSOC samples (100). They reported variable scoring in some samples, probably caused by high signal-to-noise ratio and RAD51 heterogeneity, as the tumor areas selected for quantification as well as the final scoring were operator-dependent and suggested the importance of screening the entire specimen and selecting multiple representative tumor tissue areas when using this IF assay (100). While these data suggested that the RAD51 score assessment did not yield statistical power for strong conclusions on features impacting interobserver variability, they do support its robustness.

Setting up an IHC-based RAD51 foci method would be interesting, as IHC is routinely adopted in all pathology laboratories. Some attempts have been made, but validation is still required (62, 101).

RAD51 heterogeneity within the same tissue or different biopsies of the same patient can be particularly challenging when the variable scoring surrounds the established HRD cut-off. A solution would be the acquisition of an automated system to acquire the entire tissue image for digital analysis. This would be crucial to avoid a lack of recognition of HRP clones that have the potential to drive acquired resistance to therapy. In fact, the RAD51 test is able to predict PARPi resistance by recognizing RAD51-positive cells in BRCA1/2-mutated samples where revertant mutations occur in *BRCA1/2* genes (102). As already pointed out, RAD51 test robustness can be impacted by the fixation method and the amount of proliferating tumor cells, especially in small biopsies. Finally, the validation of the threshold able to cluster HRD from HRP tumors remains challenging due to the lack of a reference method to define HRD. An attempt to calibrate the method has been described by Van Wijk et al., who analyzed two different tumor types (endometrial and epithelial ovarian cancer) using FFPE-RAD51 and the HRD-RECAP test (87). The authors established optimal parameters for RAD51 foci number (≥ 2) and HRD threshold (15%) and found, respectively, 90% and 87% test sensitivities (87).

Considering all the results obtained until now, the RAD51 foci test is an attractive, low-cost alternative to DNA-based HRD tests. Once validated and correctly integrated into clinical practice, the RAD51 test will definitely help tailor personalized treatment.

### 4.2 DNA fiber assay

Besides HR restoration, several other determinants of resistance to PARPi have been described, including alterations affecting the replication fork protection machinery (103). PARP inhibition causes dysregulation of replicative forks due to PARP-trapping, which, in HRD cells, enhances replication stress, fork collapse, and eventually induces cell death (104). BRCA proteins and RAD51 have roles outside HR, being involved in the protection of replication fork (RF) under replicative stress conditions (105), preventing RF stalling and uncontrolled RF degradation from DNA nucleases (105–107). These mechanisms have been shown in *in vitro* experiments where, in *BRCA*-deficient cells, RAD51 filaments are not stabilized and do not block the nucleolytic activity of MRE11 nuclease, which degrades the ssDNA with the formation of a shorter new strand, which causes RF collapse and is associated with PARPi sensitivity. Alterations affecting the recruitment of these nucleases to stalled RF or defective RF degradation processes have been associated with platinum and PARPi resistance in *BRCA* mutant cell lines (101, 103). RF dynamics can be studied functionally with the DNA fiber assay (108). A fiber assay visualizes the RF degradation process by an IF-based approach in *in vitro* cultures after the incorporation of two different labeled thymidine analogs: iododeoxyuridine (IdU) and chlorodeoxyuridine (CldU) (**Figure 1**) (109). RF degradation has been associated with sensitivity to chemotherapy in *BRCA*-deficient tumors, while RF protection has been associated with acquired resistance (103). Using different labeling schemes, it is possible to study several replication parameters, such as the speed of RF, the frequency of replication fork stalling, or the number of new firing origins (109). Hill et al. applied the fiber assay to HGSOC organoids treated with hydroxyurea, a RF stalling agent, and then evaluated whether tumor cells were able to protect the stalled RF from degradation (101). They observed that 61% of 33 HGSOC organoids studied had unstable RF, a condition associated with lack of RF-protecting proteins (i.e., BRCA or RAD51) and increased sensitivity to platinum drugs and PARPi. They demonstrated that 13 out of the 17 RF unstable organoids were responsive to carboplatin, and 10 out if 11 with stable RF were resistant. Regarding olaparib, two olaparib-sensitive organoids had unstable RF, while the other 15 olaparib-resistant also displayed an unstable RF but were also positive to RAD51 foci test, suggesting an HRP phenotype (101). These findings support RF instability may be a surrogate marker for platinum, but not PARPi sensitivity (101).

Preclinical evidence suggests that PARPi favors an acceleration of the RF speed and lowers the number of stalled RF in *BRCA1*-mutated cells (110). The fiber assay can help in understanding the possible mechanisms of PARPi resistance but relies on the use of fresh material and requires an *in vitro* treatment, rendering its clinical translatability difficult.

## 5 Machine learning and artificial intelligence for HRD recognition

All women with HGSOC should get access to HRD screening platforms regardless of their geographical localization and socioeconomic condition, as recently suggested by different guidelines (111). It has been reported that testing all the patients and their first-degree relatives would respectively drop the risk of breast and ovarian cancer by 20% and 55% (111). The main obstacle is represented by the high costs of genetic HRD tests, which are in most cases not refundable, thus prompting the development of more affordable strategies.

In recent years, AI has aided clinicians in improving diagnosis, i.e., by analyzing digital histopathological images (112). Indeed, AI can be trained to recognize special properties of the images starting from a large set of input data (113). Machine learning (ML) models, such as random forest, support vector machines (SVM), and linear/logistic regression, rely on supervised training, so both input data and corresponding labels are provided for the model to learn classification criteria, and the prediction of the model gets more accurate as the internal parameters are iteratively updated. External validation on a different dataset is required to determine the model’s performance and generalizability across diverse settings (114). In the case of pathology, images have to be previously annotated by expert pathologists in a laborious and time-consuming process (115). In contrast, deep learning (DL) models are trained in a nonsupervised way, so image annotation is not required as the model is able to extract specific patterns from the input dataset and use them to achieve sample stratification (116). This is the case of convolutional neural networks (CNNs), which are generally more powerful than ML models as they can get much more information from a single histopathology image but require a larger input dataset for the training. Also, as neural networks come up with more complex classification criteria, they are much harder to interpret, thus precluding the possibility of understanding the reasons for sample stratification, which would be important, especially in the oncology field (116). Features that AI is able to extract from digital images are numerical values, which may be the actual pixel values, edge strengths, variation in pixel values in a region, or other values computable from pixels. Also, nonpixel information such as age, gender, or test results can contribute to improve the stratification performance (113).

ML and DL models could be trained to predict the HRD status, as well as the presence of *BRCA1/2* mutations, by providing an input dataset including tumor samples and the related genomic test results during the training process. As the prediction mainly relies on hematoxylin and eosin (HE)-stained tumor slides, the underlying idea is that both *BRCA1/2* mutations and HR deficiency have phenotypical consequences at a histological level that can be detected from digital images (117). The major problem with whole slide images (WSI) is that they are too large to be directly processed by CNNs, so they are generally segmented into smaller tiles of a defined resolution. After appropriate annotation, the model learns and selects only those tiles containing tumoral areas whose features are expected to correlate with the presence of molecular alterations. This approach of multiple instance learning (MIL) is usually coupled with an attention approach in which the model learns how much a tile contributes to the final classification so that the tiles are scored and eventually aggregated in a weighted manner based on their contribution (115). Loeffler et al. explored the possibility of predicting the tumor HRD status based only on routine HE histology images in 10 cancer types by using an attention-weighted MIL approach (118). HRD-positive samples were conventionally designated by a GIS ≥ 42 according to Myriad test criteria. The HRD prediction model was internally validated only in three tumor types: endometrium, pancreas, and lung, with an area under the receiver operating characteristic [AUROC—a parameter indicating the accuracy of the predicting model; the closer the AUROC is to 1, the better the performance of the model (119)] of 0.79, 0.58, and 0.66, respectively. Predictions generalized well to an external cohort, providing AUROCs of 0.93, 0.81, and 0.73, respectively, for the different tumor types. In addition, they used a HRD classifier trained on breast cancer (yielding an AUROC of 0.78 in internal validation) for predicting HRD in endometrial, prostate, and pancreatic cancer, providing AUROCs of 0.87, 0.84, and 0.67, respectively, thus supporting that a HRD-like phenotype is shared across different tumor entities (118). Lazard et al. pointed out the difficulty of interpreting DL classification criteria, which prevents the identification of possible confounding factors leading to a biased stratification (117). These authors used a large retrospective cohort of 714 luminal and TNBC patients with a genomically defined HR status from a single cancer center. The neural network was trained to predict HRD from HE digital images using the MIL approach, and the model’s performance was demonstrated to be higher than those models trained on pan-cancer datasets (AUC = 0.88 vs. AUC = 0.71). By applying the same classification model to a tile level, they were able to obtain a morphological map of HRD throughout the whole tissue slide and were able to detect specific morphological patterns related to HRD-positive areas of the tumor (117).

The analysis of digital pathological images could also allow prognosis stratification. Wu et al. applied a DL model to all the samples from The Cancer Genome Atlas Program (TCGA)-ovarian cancer cohort obtaining a risk-score, that eventually correlated with tumor HRD status, tumor response to platinum-based chemotherapy, and patients’ overall survival (112).

AI can be used to develop improved algorithms to predict tumors’ HRD status based on genomic data. This is the case of GIInger, a cost-effective and easy-to-implement method to identify HRD tumors (57). The authors developed a deep learning-based model able to extract valuable information from low-pass WGS (lpWGS), whose depth of sequencing (1×) allowed them to detect mainly LST, compared to much deeper WGS approaches, but also TAI and LOH were needed to determine the GI score. However, the deeper the coverage of the sequencing, the more expensive the process, and so the harder could be its broad implementation in oncology patients.

Pozzorini et al. (57) validated GIInger using lpWGS data from fresh frozen breast cancer samples and found a strong correlation between the HRD status predicted by the model and the HRDetect score of the samples. They also evaluated GIInger’s analytical performance on FFPE OC samples in a multicenter setting, obtaining robust and reproducible results across different laboratories and high concordance with the reference method (57). GIInger was also tested in a subset of PAOLA-1/ENGOT-ov25 trial patients (EHEI), and predicted HRD status and drug response with higher accuracy than alternative tests, suggesting GIInger clinical utility (57). Albitar et al. (120) trained their ML model based on copy number alteration (CNA) identification on a panel of 434 genes aimed at distinguishing *BRCA1/2* mutated from *BRCA1/2* wt tumors. The model was used to stratify a dataset composed of 31 ovarian or breast cancers with confirmed *BRCA1/2* mutations and 84 cases negative for mutations in BRCA1/2 or any of the 12 selected genes implicated in HR, providing an AUROC of 0.984, a sensitivity of 90%, and a specificity of 98%. Interestingly, since the presence of *BRCA1/2* mutations is the major cause of HRD, they used their ML model to predict the HRD status of 124 ovarian or breast cancers without mutations in any of the HR genes and 114 cancers with mutations in one of the genes involved in HR. They observed that in both groups, the system defined HRD-positive tumors, suggesting a shared *BRCAness* phenotype. Unfortunately, a clinical correlation with treatment and survival of the patients was missing (120).

Deep learning also provides the opportunity to integrate multi-omics data to better predict HRD, not only on genetic alterations but also on transcriptional and epigenetic levels. Zhang et al. (121) developed a Multi-Omics Integrative Deep-learning framework to better predict HRD-positive tumors based on information from RNA-seq, miRNA-seq, DNA methylation, and somatic mutations (121). The model was trained on a dataset comprising 551 samples, including 351 true samples (204 HRD and 147 HRP) from TCGA-OV cohort and 200 samples (100 HRD and 100 HRP) from data augmentation to avoid overfitting caused by insufficient training dataset. The MODeepHRD was trained to predict HRD status from single omics, then combined to get the final prediction probability for HRD-positive phenotype (AUC of 0.88, accuracy of 0.88, sensitivity of 0.85, and specificity of 0.9). Interestingly, when the model was trained on coupled omics, e.g., transcriptome/mutation or transcriptome/methylation, either its sensitivity or specificity increased. A good correlation between MODeepHRD prediction and OS and DFS in TCGA-OV cohort was found (121). Model validation was performed using 2,070 OC samples from 21 microarray datasets combined, where both platinum-based chemotherapy response and survival were improved in HRD-positive predicted tumors compared to HRD-negative tumors. The predicting performance of the model was compared with already existing ML models as well as previously reported HRD detection methods, yielding a higher AUC and showing higher sensitivity in predicting HRD based on mutations in HR-related genes in different TCGA cohorts, supporting the feasibility and superiority of the MODeepHRD model to predict HRD phenotype in gynecological cancers. These data support the MODeepHRD model as a reliable tool for accurately screening HRD phenotype and guiding therapeutic decisions (121).

In conclusion, AI is becoming fundamental for the computer-based biomarker discovery field as it allows for the management and processing of huge amounts of data coming from either digital images and/or molecular omics approaches. Further improvement and adoption of more sophisticated ML and DL models show great promise for the future of biomarker discovery (116).

## 6 Conclusions/future perspectives

**Figure 2:**
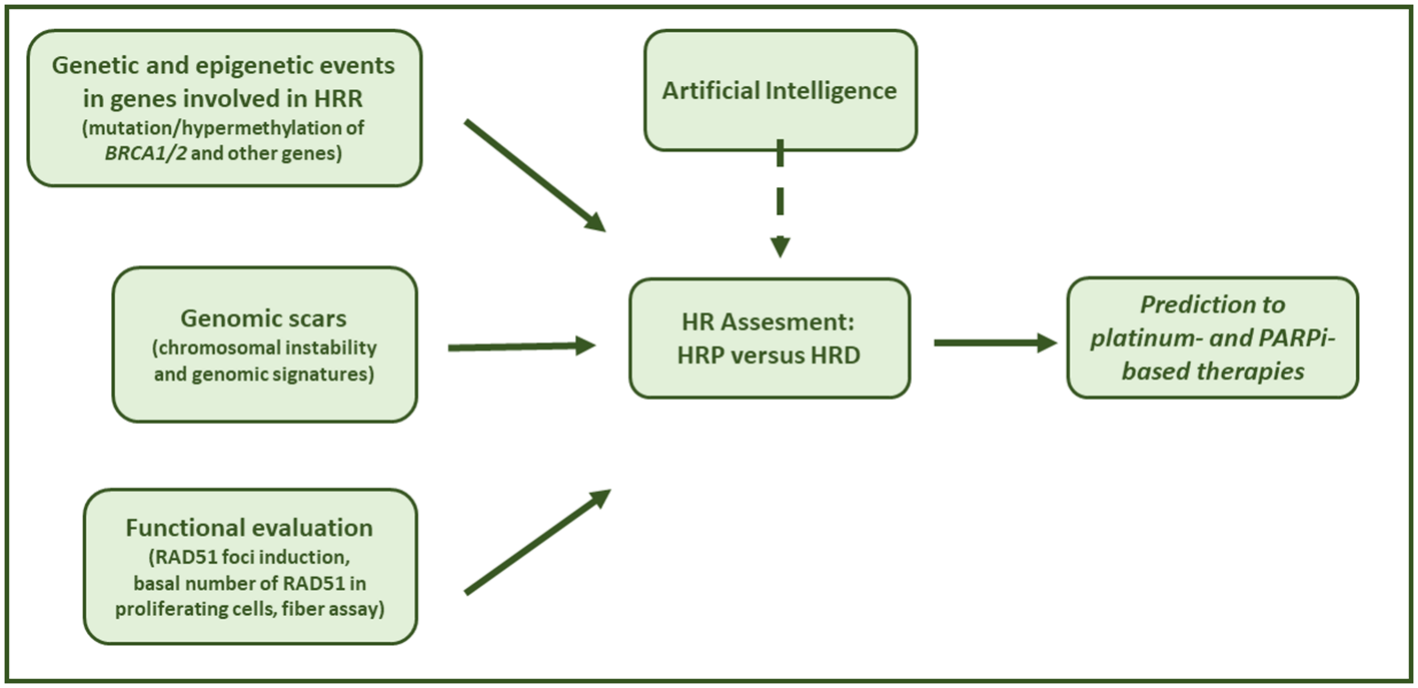
Assessing HRD in the clinical setting. Graphical summary of HRD testing solutions to assess cancer HRR phenotype in order to predict response to platinum chemotherapy and PARPi-based therapy.

The HRD phenotype is characterized by the functional inactivation of HR and the inability to repair DNA-DSBs in an error-free manner. Indeed, HR repairs the DNA-DSBs using the sister chromatid as a template, with no modification/loss of the genetic material (122). The lack of inactivation of the pathway due to genetic and epigenetic events that disable the function of HR genes leads to the repair of DNA-DSBs by error-prone pathways (i.e. NHEJ, MHEJ) with the accumulation of genetic damages (named scars), favoring genetic instability and tumorigenesis (123, 124). Recently, the synthetic lethal interaction between HRD and inhibition of PARP has been shown to have therapeutic value in a clinical setting. These considerations are important in HGSOC, half of which have been reported to be HR-deficient. Indeed, both the National Comprehensive Cancer Network (NCCN) and the ESMO guidelines recommend HR testing of somatic and germline mutations in *BRCA1/2* and in HR-related genes, and/or genomic scars (12, 13) to identify HR deficiency. Functional tests have been developed, but they still require to be validated in the clinical setting (**Figure 2**).

In this review, the different HRD tests developed over the last decade were summarized, and their potential as well as their limits were discussed. The implementation of these tests in the real world is and will be a real challenge in the next future. While robust implementation studies have been done with *BRCA* germline and somatic mutations, as these were first available, much still has to be done with genomic scar assays. Some of these have been companion diagnostics, instrumental in the clinical development of PARPi, but their wide implementation has not yet occurred due to several reasons, including economic aspects (i.e., national health policies that do not provide reimbursement for testing), centralization of testing, and long lead times. Among new genomic tests, the most promising are the ones deriving from researchers from the EHEI, based on new in-house HRD tests based on mutational signatures and patterns of genomic scars (53). Interestingly, these tests showed high concordance with Myriad MyChoice CDx in the PAOLA-1/ENGOT-ov25 trial tumor samples and low failure rates, but require validation studies in new patients’ cohorts. The role of AI is increasing in the diagnostic and therapeutic journey of oncologic patients and will likely play a fundamental role in the definition of tumor HR status (HRD or HRP), as recent literature suggests (125, 126). HRD functional tests hold great promise, being able to capture not only the tumor history (genomic scar) but possibly its actual HR status (127). In addition, these latter tests will likely predict and/or anticipate the resistance to both platinum drugs and PARPi due to the reacquisition of a functional HR repair, the most diffused mechanism of resistance reported in the clinic (128). Interestingly, the recent advancements in proteomics technologies, including mass spectrometry and protein array analyses, will be valuable complementary methods to study HR in the near future and could help not only in patient stratification to tailor treatment but also to study adaptive response to therapy and potentially intercept drug resistance (129, 130).

## References

[1] Saani I Raj N Sood R Ansari S Mandviwala HA Sanchez E. Clinical challenges in the management of Malignant ovarian germ cell tumours. Int J Environ Res Public Health. (2023) 20:6089. doi: 10.3390/ijerph20126089 37372675 PMC10298722

[2] Lheureux S Gourley C Vergote I Oza AM. Epithelial ovarian cancer. Lancet. (2019) 393:1240–53. doi: 10.1016/S0140-6736(18)32552-2 30910306

[3] Lheureux S Lai Z Dougherty BA Runswick S Hodgson DR Timms KM. Long-term responders on olaparib maintenance in high-grade serous ovarian cancer: clinical and molecular characterization. Clin Cancer Res. (2017) 23:4086–94. doi: 10.1158/1078-0432.CCR-16-2615 28223274

[4] Jayson GC Kohn EC Kitchener HC Ledermann JA. Ovarian cancer. Lancet. (2014) 384:1376–88. doi: 10.1016/S0140-6736(13)62146-7 24767708

[5] Konstantinopoulos PA Ceccaldi R Shapiro GI D’Andrea AD. Homologous recombination deficiency: exploiting the fundamental vulnerability of ovarian cancer. Cancer Discovery. (2015) 5:1137–54. doi: 10.1158/2159-8290.CD-15-0714 PMC4631624 26463832

[6] Aliyuda F Moschetta M Ghose A Sofia Rallis K Sheriff M Sanchez E. Advances in ovarian cancer treatment beyond PARP inhibitors. Curr Cancer Drug Targets. (2023) 23:433–46. doi: 10.2174/1568009623666230209121732 36757037

[7] Lord CJ Ashworth A. BRCAness revisited. Nat Rev Cancer. (2016) 16:110–20. doi: 10.1038/nrc.2015.21 26775620

[8] Norquist BM Brady MF Harrell MI Walsh T Lee MK Gulsuner S. Mutations in homologous recombination genes and outcomes in ovarian carcinoma patients in GOG 218: an NRG oncology/gynecologic oncology group study. Clin Cancer Res. (2018) 24:777–83. doi: 10.1158/1078-0432.CCR-17-1327 PMC5815909 29191972

[9] Ter Brugge P Moser SC Bièche I Kristel P Ibadioune S Eeckhoutte A. Homologous recombination deficiency derived from whole-genome sequencing predicts platinum response in triple-negative breast cancers. Nat Commun. (2023) 14:1958. doi: 10.1038/s41467-023-37537-2 37029129 PMC10082194

[10] Chandrasekaran A Elias KM. Synthetic lethality in ovarian cancer. Mol Cancer Ther. (2021) 20:2117–28. doi: 10.1158/1535-7163.MCT-21-0500 PMC8571039 34518297

[11] Konstantinopoulos PA Spentzos D Karlan BY Taniguchi T Fountzilas E Francoeur N. Gene expression profile of BRCAness that correlates with responsiveness to chemotherapy and with outcome in patients with epithelial ovarian cancer. J Clin Oncol. (2010) 28:3555–61. doi: 10.1200/JCO.2009.27.5719 PMC2917311 20547991

[12] Armstrong DK Alvarez RD Backes FJ Bakkum-Gamez JN Barroilhet L Behbakht K. NCCN guidelines® Insights: ovarian cancer, version 3.2022. J Natl Compr Canc Netw. (2022) 20:972–80. doi: 10.6004/jnccn.2022.0047u 36075393

[13] Ledermann JA Matias-Guiu X Amant F Concin N Davidson B Fotopoulou C. ESGO-ESMO-ESP consensus conference recommendations on ovarian cancer: pathology and molecular biology and early, advanced and recurrent disease. Ann Oncol. (2024) 35:248–66. doi: 10.1016/j.annonc.2023.11.015 38307807

[14] Mangogna A Munari G Pepe F Maffii E Giampaolino P Ricci G. Homologous recombination deficiency in ovarian cancer: from the biological rationale to current diagnostic approaches. J Pers Med. (2023) 13:284. doi: 10.3390/jpm13020284 36836518 PMC9968181

[15] Hoppe MM Sundar R Tan DSP Jeyasekharan AD. Biomarkers for homologous recombination deficiency in cancer. J Natl Cancer Inst. (2018) 110:704–13. doi: 10.1093/jnci/djy085 29788099

[16] Ali U Vungarala S Tiriveedhi V. Genomic features of homologous recombination deficiency in breast cancer: impact on testing and immunotherapy. Genes. (2024) 15:162. doi: 10.3390/genes15020162 38397152 PMC10887603

[17] Nichols CA Gibson WJ Brown MS Kosmicki JA Busanovich JP Wei H. Loss of heterozygosity of essential genes represents a widespread class of potential cancer vulnerabilities. Nat Commun. (2020) 11:2517. doi: 10.1038/s41467-020-16399-y 32433464 PMC7239950

[18] Abkevich V Timms KM Hennessy BT Potter J Carey MS Meyer LA. Patterns of genomic loss of heterozygosity predict homologous recombination repair defects in epithelial ovarian cancer. Br J Cancer. (2012) 107:1776–82. doi: 10.1038/bjc.2012.451 PMC3493866 23047548

[19] Birkbak NJ Wang ZC Kim J-Y Eklund AC Li Q Tian R. Telomeric allelic imbalance indicates defective DNA repair and sensitivity to DNA-damaging agents. Cancer Discovery. (2012) 2:366–75. doi: 10.1158/2159-8290.CD-11-0206 PMC3806629 22576213

[20] Popova T Manié E Rieunier G Caux-Moncoutier V Tirapo C Dubois T. Ploidy and large-scale genomic instability consistently identify basal-like breast carcinomas with BRCA1/2 inactivation. Cancer Res. (2012) 72:5454–62. doi: 10.1158/0008-5472.CAN-12-1470 22933060

[21] Timms KM Abkevich V Hughes E Neff C Reid J Morris B. Association of BRCA1/2defects with genomic scores predictive of DNA damage repair deficiency among breast cancer subtypes. Breast Cancer Res. (2014) 16:475. doi: 10.1186/s13058-014-0475-x 25475740 PMC4308910

[22] Telli ML Timms KM Reid J Hennessy B Mills GB Jensen KC. Homologous recombination deficiency (HRD) score predicts response to platinum-containing neoadjuvant chemotherapy in patients with triple-negative breast cancers. Clin Cancer Res. (2016) 22:3764–73. doi: 10.1158/1078-0432.CCR-15-2477 PMC6773427 26957554

[23] Ngoi NYL Tan DSP. The role of homologous recombination deficiency testing in ovarian cancer and its clinical implications: do we need it? ESMO Open. (2021) 6:100144. doi: 10.1016/j.esmoop.2021.100144 34015643 PMC8141874

[24] Stronach EA Paul J Timms KM Hughes E Brown K Neff C. Biomarker assessment of HR deficiency, tumor BRCA1/2 mutations, and CCNE1 copy number in ovarian cancer: associations with clinical outcome following platinum monotherapy. Mol Cancer Res. (2018) 16:1103–11. doi: 10.1158/1541-7786.MCR-18-0034 29724815

[25] Pellegrino B Mateo J Serra V Balmaña J. Controversies in oncology: are genomic tests quantifying homologous recombination repair deficiency (HRD) useful for treatment decision making? ESMO Open. (2019) 4:e000480. doi: 10.1136/esmoopen-2018-000480 31231558 PMC6555601

[26] Coleman RL Oza AM Lorusso D Aghajanian C Oaknin A Dean A. Rucaparib maintenance treatment for recurrent ovarian carcinoma after response to platinum therapy (ARIEL3): a randomised, double-blind, placebo-controlled, phase 3 trial. Lancet. (2017) 390:1949–61. doi: 10.1016/S0140-6736(17)32440-6 PMC5901715 28916367

[27] Ledermann JA Oza AM Lorusso D Aghajanian C Oaknin A Dean A. Rucaparib for patients with platinum-sensitive, recurrent ovarian carcinoma (ARIEL3): post-progression outcomes and updated safety results from a randomised, placebo-controlled, phase 3 trial. Lancet Oncol. (2020) 21:710–22. doi: 10.1016/S1470-2045(20)30061-9 PMC8210534 32359490

[28] Cordani N Bianchi T Ammoni LC Cortinovis DL Cazzaniga ME Lissoni AA. An overview of PARP resistance in ovarian cancer from a molecular and clinical perspective. Int J Mol Sci. (2023) 24:11890. doi: 10.3390/ijms241511890 37569269 PMC10418869

[29] González-Martín A Pothuri B Vergote I DePont Christensen R Graybill W Mirza MR. Niraparib in patients with newly diagnosed advanced ovarian cancer. N Engl J Med. (2019) 381:2391–402. doi: 10.1056/NEJMoa1910962 31562799

[30] Ray-Coquard I Pautier P Pignata S Pérol D González-Martín A Berger R. Olaparib plus bevacizumab as first-line maintenance in ovarian cancer. New Engl J Med. (2019) 381:2416–28. doi: 10.1056/NEJMoa1911361 31851799

[31] Coleman RL Fleming GF Brady MF Swisher EM Steffensen KD Friedlander M. Veliparib with first-line chemotherapy and as maintenance therapy in ovarian cancer. New Engl J Med. (2019) 381:2403–15. doi: 10.1056/NEJMoa1909707 PMC6941439 31562800

[32] Monk BJ Parkinson C Lim MC O’Malley DM Oaknin A Wilson MK. A randomized, phase III trial to evaluate rucaparib monotherapy as maintenance treatment in patients with newly diagnosed ovarian cancer (ATHENA–MONO/GOG-3020/ENGOT-ov45). JCO. (2022) 40:3952–64. doi: 10.1200/JCO.22.01003 PMC9746782 35658487

[33] Moore K Colombo N Scambia G Kim B-G Oaknin A Friedlander M. Maintenance olaparib in patients with newly diagnosed advanced ovarian cancer. N Engl J Med. (2018) 379:2495–505. doi: 10.1056/NEJMoa1810858 30345884

[34] Banerjee S Moore KN Colombo N Scambia G Kim B-G Oaknin A. Maintenance olaparib for patients with newly diagnosed advanced ovarian cancer and a BRCA mutation (SOLO1/GOG 3004): 5-year follow-up of a randomised, double-blind, placebo-controlled, phase 3 trial. Lancet Oncol. (2021) 22:1721–31. doi: 10.1016/S1470-2045(21)00531-3 34715071

[35] Ledermann J Harter P Gourley C Friedlander M Vergote I Rustin G. Olaparib maintenance therapy in platinum-sensitive relapsed ovarian cancer. New Engl J Med. (2012) 366:1382–92. doi: 10.1056/NEJMoa1105535 22452356

[36] Ledermann J Harter P Gourley C Friedlander M Vergote I Rustin G. Olaparib maintenance therapy in patients with platinum-sensitive relapsed serous ovarian cancer: a preplanned retrospective analysis of outcomes by BRCA status in a randomised phase 2 trial. Lancet Oncol. (2014) 15:852–61. doi: 10.1016/S1470-2045(14)70228-1 24882434

[37] Matulonis UA Harter P Gourley C Friedlander M Vergote I Rustin G. Olaparib maintenance therapy in patients with platinum-sensitive, relapsed serous ovarian cancer and a BRCA mutation: Overall survival adjusted for postprogression poly(adenosine diphosphate ribose) polymerase inhibitor therapy. Cancer. (2016) 122:1844–52. doi: 10.1002/cncr.29995 27062051

[38] Pujade-Lauraine E Ledermann JA Selle F Gebski V Penson RT Oza AM. Olaparib tablets as maintenance therapy in patients with platinum-sensitive, relapsed ovarian cancer and a BRCA1/2 mutation (SOLO2/ENGOT-Ov21): a double-blind, randomised, placebo-controlled, phase 3 trial. Lancet Oncol. (2017) 18:1274–84. doi: 10.1016/S1470-2045(17)30469-2 28754483

[39] Poveda A Floquet A Ledermann JA Asher R Penson RT Oza AM. Final overall survival (OS) results from SOLO2/ENGOT-ov21: A phase III trial assessing maintenance olaparib in patients (pts) with platinum-sensitive, relapsed ovarian cancer and a BRCA mutation. JCO. (2020) 38:6002–2. doi: 10.1200/JCO.2020.38.15_suppl.6002

[40] Mirza MR Monk BJ Herrstedt J Oza AM Mahner S Redondo A. Niraparib maintenance therapy in platinum-sensitive, recurrent ovarian cancer. N Engl J Med. (2016) 375:2154–64. doi: 10.1056/NEJMoa1611310 27717299

[41] Maiorano MFP Maiorano BA Biancofiore A Cormio G Maiello E. Niraparib and advanced ovarian cancer: A beacon in the non-BRCA mutated setting. Pharm (Basel). (2023) 16:1261. doi: 10.3390/ph16091261 PMC10536506 37765068

[42] Cadoo K Simpkins F Mathews C Liu YL Provencher D McCormick C. Olaparib treatment for platinum-sensitive relapsed ovarian cancer by BRCA mutation and homologous recombination deficiency status: Phase II LIGHT study primary analysis. Gynecol Oncol. (2022) 166:425–31. doi: 10.1016/j.ygyno.2022.06.017 PMC9909678 35803835

[43] Moore KN Secord AA Geller MA Miller DS Cloven N Fleming GF. Niraparib monotherapy for late-line treatment of ovarian cancer (QUADRA): a multicentre, open-label, single-arm, phase 2 trial. Lancet Oncol. (2019) 20:636–48. doi: 10.1016/S1470-2045(19)30029-4 30948273

[44] Timms KM Mills GB Perry M Gutin A Lanchbury J Brown R. Comparison of genomic instability test scores used for predicting PARP activity in ovarian cancer. JCO. (2020) 38:1586–6. doi: 10.1200/JCO.2020.38.15_suppl.1586

[45] Gonzalez D Stenzinger A. Homologous recombination repair deficiency (HRD): From biology to clinical exploitation. Genes Chromosomes Cancer. (2021) 60:299–302. doi: 10.1002/gcc.22939 33486842

[46] Quesada S Fabbro M Solassol J. Toward more comprehensive homologous recombination deficiency assays in ovarian cancer, part 1: technical considerations. Cancers (Basel). (2022) 14:1132. doi: 10.3390/cancers14051132 35267439 PMC8909526

[47] Wagener-Ryczek S Merkelbach-Bruse S Siemanowski J. Biomarkers for homologous recombination deficiency in cancer. J Pers Med. (2021) 11:612. doi: 10.3390/jpm11070612 34203281 PMC8304859

[48] Denkert C Romey M Swedlund B Hattesohl A Teply-Szymanski J Kommoss S. Homologous recombination deficiency as an ovarian cancer biomarker in a real-world cohort: validation of decentralized genomic profiling. J Mol Diagnostics. (2022) 24:1254–63. doi: 10.1016/j.jmoldx.2022.09.004 36191839

[49] Heitz F Ataseven B Staniczok C Denkert C Rhiem K Hahnen E. Implementing HRD testing in routine clinical practice on patients with primary high-grade advanced ovarian cancer. Cancers (Basel). (2023) 15:818. doi: 10.3390/cancers15030818 36765776 PMC9913091

[50] Colombo N Sessa C du Bois A Ledermann J McCluggage WG McNeish I. ESMO-ESGO consensus conference recommendations on ovarian cancer: pathology and molecular biology, early and advanced stages, borderline tumours and recurrent disease†. Ann Oncol. (2019) 30:672–705. doi: 10.1093/annonc/mdz062 31046081

[51] Miller RE Leary A Scott CL Serra V Lord CJ Bowtell D. ESMO recommendations on predictive biomarker testing for homologous recombination deficiency and PARP inhibitor benefit in ovarian cancer. Ann Oncol. (2020) 31:1606–22. doi: 10.1016/j.annonc.2020.08.2102 33004253

[52] Vanstapel FJLA Orth M Streichert T Capoluongo ED Oosterhuis WP Çubukçu HC. ISO 15189 is a sufficient instrument to guarantee high-quality manufacture of laboratory developed tests for in-house-use conform requirements of the European *In-Vitro*-Diagnostics Regulation. Clin Chem Lab Med. (2023) 61:608–26. doi: 10.1515/cclm-2023-0045 36716120

[53] Pujade-Lauraine E Christinat Y D’incalci M Schouten P Buisson A Heukamp L. Homologous recombination deficiency testing in advanced ovarian cancer: description of the ENGOT HRD European initiative. Ovarian Cancer. (2021), A208–8. doi: 10.1136/ijgc-2021-ESGO.356

[54] Capoluongo ED Pellegrino B Arenare L Califano D Scambia G Beltrame L. Alternative academic approaches for testing homologous recombination deficiency in ovarian cancer in the MITO16A/MaNGO-OV2 trial. ESMO Open. (2022) 7:100585. doi: 10.1016/j.esmoop.2022.100585 36156447 PMC9512829

[55] Christinat Y Ho L Clément S Genestie C Sehouli J Cinieri S. Normalized LST is an efficient biomarker for homologous recombination deficiency and olaparib response in ovarian carcinoma. JCO Precis Oncol. (2023) 7:e2200555. doi: 10.1200/PO.22.00555 37364234 PMC10581603

[56] Loverix L Vergote I Busschaert P Vanderstichele A Venken T Boeckx B. PARP inhibitor predictive value of the Leuven HRD test compared with Myriad MyChoice CDx PLUS HRD on 468 ovarian cancer patients from the PAOLA-1/ENGOT-ov25 trial. Eur J Cancer. (2023) 188:131–9. doi: 10.1016/j.ejca.2023.04.020 37245441

[57] Pozzorini C Andre G Coletta T Buisson A Bieler J Ferrer L. GIInger predicts homologous recombination deficiency and patient response to PARPi treatment from shallow genomic profiles. Cell Rep Med. (2023) 4:101344. doi: 10.1016/j.xcrm.2023.101344 38118421 PMC10772634

[58] Schouten PC Richters L Vis DJ Kommoss S van Dijk E Ernst C. Ovarian cancer-specific BRCA-like copy-number aberration classifiers detect mutations associated with homologous recombination deficiency in the AGO-TR1 trial. Clin Cancer Res. (2021) 27:6559–69. doi: 10.1158/1078-0432.CCR-21-1673 PMC9401539 34593530

[59] Willing E-M Vollbrecht C Vössing C Weist P Schallenberg S Herbst JM. Development of the NOGGO GIS v1 assay, a comprehensive hybrid-capture-based NGS assay for therapeutic stratification of homologous repair deficiency driven tumors and clinical validation. Cancers (Basel). (2023) 15:3445. doi: 10.3390/cancers15133445 37444554 PMC10341077

[60] Callens C Rodrigues M Briaux A Frouin E Eeckhoutte A Pujade-Lauraine E. Shallow whole genome sequencing approach to detect Homologous Recombination Deficiency in the PAOLA-1/ENGOT-OV25 phase-III trial. Oncogene. (2023) 42:3556–63. doi: 10.1038/s41388-023-02839-8 PMC10673712 37945748

[61] Davies H Glodzik D Morganella S Yates LR Staaf J Zou X. HRDetect is a predictor of BRCA1 and BRCA2 deficiency based on mutational signatures. Nat Med. (2017) 23:517–25. doi: 10.1038/nm.4292 PMC5833945 28288110

[62] Chopra N Tovey H Pearson A Cutts R Toms C Proszek P. Homologous recombination DNA repair deficiency and PARP inhibition activity in primary triple negative breast cancer. Nat Commun. (2020) 11:2662. doi: 10.1038/s41467-020-16142-7 32471999 PMC7260192

[63] Durinikova E Reilly NM Buzo K Mariella E Chilà R Lorenzato A. Targeting the DNA damage response pathways and replication stress in colorectal cancer. Clin Cancer Res. (2022) 28:3874–89. doi: 10.1158/1078-0432.CCR-22-0875 PMC9433963 35881546

[64] Leman R Muller E Legros A Goardon N Chentli I Atkinson A. Validation of the clinical use of GIScar, an academic-developed genomic instability score predicting sensitivity to maintenance olaparib for ovarian cancer. Clin Cancer Res. (2023) 29:4419–29. doi: 10.1158/1078-0432.CCR-23-0898 PMC10618649 37756555

[65] Krumm N Khasnavis NS Radke M Banda K Davies HR Pennil C. Diagnosis of ovarian carcinoma homologous recombination DNA repair deficiency from targeted gene capture oncology assays. JCO Precis Oncol. (2023) 7:e2200720. doi: 10.1200/PO.22.00720 37196218 PMC10309534

[66] Scaglione GL Pignata S Pettinato A Paolillo C Califano D Scandurra G. Homologous recombination deficiency (HRD) scoring, by means of two different shallow whole-genome sequencing pipelines (sWGS), in ovarian cancer patients: A comparison with myriad myChoice assay. Int J Mol Sci. (2023) 24:17095. doi: 10.3390/ijms242317095 38069422 PMC10707691

[67] Hakkaart C Pearson JF Marquart L Dennis J Wiggins GAR Barnes DR. Copy number variants as modifiers of breast cancer risk for BRCA1/BRCA2 pathogenic variant carriers. Commun Biol. (2022) 5:1061. doi: 10.1038/s42003-022-03978-6 36203093 PMC9537519

[68] Eeckhoutte A Houy A Manié E Reverdy M Bièche I Marangoni E. ShallowHRD: detection of homologous recombination deficiency from shallow whole genome sequencing. Bioinformatics. (2020) 36:3888–9. doi: 10.1093/bioinformatics/btaa261 PMC7320600 32315385

[69] Guffanti F Alvisi MF Anastasia A Ricci F Chiappa M Llop-Guevara A. Basal expression of RAD51 foci predicts olaparib response in patient-derived ovarian cancer xenografts. Br J Cancer. (2022) 126:120–8. doi: 10.1038/s41416-021-01609-1 PMC8727677 34732853

[70] Swisher EM Lin KK Oza AM Scott CL Giordano H Sun J. Rucaparib in relapsed, platinum-sensitive high-grade ovarian carcinoma (ARIEL2 Part 1): an international, multicentre, open-label, phase 2 trial. Lancet Oncol. (2017) 18:75–87. doi: 10.1016/S1470-2045(16)30559-9 27908594

[71] Kondrashova O Nguyen M Shield-Artin K Tinker AV Teng NNH Harrell MI. Secondary somatic mutations restoring RAD51C and RAD51D associated with acquired resistance to the PARP inhibitor rucaparib in high-grade ovarian carcinoma. Cancer Discovery. (2017) 7:984–98. doi: 10.1158/2159-8290.CD-17-0419 PMC5612362 28588062

[72] Tobalina L Armenia J Irving E O’Connor MJ Forment JV. A meta-analysis of reversion mutations in BRCA genes identifies signatures of DNA end-joining repair mechanisms driving therapy resistance. Ann Oncol. (2021) 32:103–12. doi: 10.1016/j.annonc.2020.10.470 33091561

[73] Bonilla B Hengel SR Grundy MK Bernstein KA. RAD51 gene family structure and function. Annu Rev Genet. (2020) 54:25–46. doi: 10.1146/annurev-genet-021920-092410 32663049 PMC7703940

[74] Mason JM Chan Y-L Weichselbaum RW Bishop DK. Non-enzymatic roles of human RAD51 at stalled replication forks. Nat Commun. (2019) 10:4410. doi: 10.1038/s41467-019-12297-0 31562309 PMC6764946

[75] Essers J Hendriks RW Wesoly J Beerens CEMT Smit B Hoeijmakers JHJ. Analysis of mouse Rad54 expression and its implications for homologous recombination. DNA Repair (Amst). (2002) 1:779–93. doi: 10.1016/S1568-7864(02)00110-6 12531026

[76] Bhattacharyya A Ear US Koller BH Weichselbaum RR Bishop DK. The breast cancer susceptibility gene BRCA1 is required for subnuclear assembly of Rad51 and survival following treatment with the DNA cross-linking agent cisplatin. J Biol Chem. (2000) 275:23899–903. doi: 10.1074/jbc.C000276200 10843985

[77] Willers H Taghian AG Luo C-M Treszezamsky A Sgroi DC Powell SN. Utility of DNA repair protein foci for the detection of putative BRCA1 pathway defects in breast cancer biopsies. Mol Cancer Res. (2009) 7:1304–9. doi: 10.1158/1541-7786.MCR-09-0149 PMC4239295 19671671

[78] Mukhopadhyay A Elattar A Cerbinskaite A Wilkinson SJ Drew Y Kyle S. Development of a functional assay for homologous recombination status in primary cultures of epithelial ovarian tumor and correlation with sensitivity to poly(ADP-ribose) polymerase inhibitors. Clin Cancer Res. (2010) 16:2344–51. doi: 10.1158/1078-0432.CCR-09-2758 20371688

[79] Mukhopadhyay A Plummer ER Elattar A Soohoo S Uzir B Quinn JE. Clinicopathological features of homologous recombination-deficient epithelial ovarian cancers: sensitivity to PARP inhibitors, platinum, and survival. Cancer Res. (2012) 72:5675–82. doi: 10.1158/0008-5472.CAN-12-0324 23066035

[80] Wohlschlegel JA Kutok JL Weng AP Dutta A. Expression of geminin as a marker of cell proliferation in normal tissues and Malignancies. Am J Pathology. (2002) 161:267–73. doi: 10.1016/S0002-9440(10)64178-8 PMC1850683 12107111

[81] Mao Z Bozzella M Seluanov A Gorbunova V. DNA repair by nonhomologous end joining and homologous recombination during cell cycle in human cells. Cell Cycle. (2008) 7:2902–6. doi: 10.4161/cc.7.18.6679 PMC2754209 18769152

[82] Graeser M McCarthy A Lord CJ Savage K Hills M Salter J. A marker of homologous recombination predicts pathologic complete response to neoadjuvant chemotherapy in primary breast cancer. Clin Cancer Res. (2010) 16:6159–68. doi: 10.1158/1078-0432.CCR-10-1027 PMC3432445 20802015

[83] Naipal KAT Verkaik NS Ameziane N van Deurzen CHM Ter Brugge P Meijers M. Functional ex vivo assay to select homologous recombination-deficient breast tumors for PARP inhibitor treatment. Clin Cancer Res. (2014) 20:4816–26. doi: 10.1158/1078-0432.CCR-14-0571 24963051

[84] Meijer TG Verkaik NS Sieuwerts AM van Riet J Naipal KAT van Deurzen CHM. Functional ex vivo assay reveals homologous recombination deficiency in breast cancer beyond BRCA gene defects. Clin Cancer Res. (2018) 24:6277–87. doi: 10.1158/1078-0432.CCR-18-0063 30139880

[85] Meijer TG Nguyen L Van Hoeck A Sieuwerts AM Verkaik NS Ladan MM. Functional RECAP (REpair CAPacity) assay identifies homologous recombination deficiency undetected by DNA-based BRCAness tests. Oncogene. (2022) 41:3498–506. doi: 10.1038/s41388-022-02363-1 PMC9232391 35662281

[86] van Wijk LM Vermeulen S Meijers M van Diest MF Ter Haar NT de Jonge MM. The RECAP test rapidly and reliably identifies homologous recombination-deficient ovarian carcinomas. Cancers (Basel). (2020) 12:2805. doi: 10.3390/cancers12102805 33003546 PMC7650677

[87] van Wijk LM Kramer CJH Vermeulen S Ter Haar NT de Jonge MM Kroep JR. The RAD51-FFPE test; calibration of a functional homologous recombination deficiency test on diagnostic endometrial and ovarian tumor blocks. Cancers (Basel). (2021) 13:2994. doi: 10.3390/cancers13122994 34203855 PMC8232577

[88] van Wijk LM Vermeulen S Ter Haar NT Kramer CJH Terlouw D Vrieling H. Performance of a RAD51-based functional HRD test on paraffin-embedded breast cancer tissue. Breast Cancer Res Treat. (2023) 202:607–16. doi: 10.1007/s10549-023-07102-y PMC10564840 37725154

[89] Meijer TG Verkaik NS van Deurzen CHM Dubbink H-J den Toom TD Sleddens HFBM. Direct ex vivo observation of homologous recombination defect reversal after DNA-damaging chemotherapy in patients with metastatic breast cancer. JCO Precis Oncol. (2019) 3:1–12. doi: 10.1200/PO.18.00268 35100677

[90] de Jonge MM Auguste A van Wijk LM Schouten PC Meijers M Ter Haar NT. Frequent homologous recombination deficiency in high-grade endometrial carcinomas. Clin Cancer Res. (2019) 25:1087–97. doi: 10.1158/1078-0432.CCR-18-1443 30413523

[91] Tumiati M Hietanen S Hynninen J Pietilä E Färkkilä A Kaipio K. A functional homologous recombination assay predicts primary chemotherapy response and long-term survival in ovarian cancer patients. Clin Cancer Res. (2018) 24:4482–93. doi: 10.1158/1078-0432.CCR-17-3770 29858219

[92] Tumiati M Hietanen S Kauppi L. Time to go functional! Determining tumors’ DNA repair capacity ex vivo. Oncotarget. (2018) 9:36826–7. doi: 10.18632/oncotarget.v9i96 PMC6305149 30627321

[93] Cruz C Castroviejo-Bermejo M Gutiérrez-Enríquez S Llop-Guevara A Ibrahim YH Gris-Oliver A. RAD51 foci as a functional biomarker of homologous recombination repair and PARP inhibitor resistance in germline BRCA-mutated breast cancer. Ann Oncol. (2018) 29:1203–10. doi: 10.1093/annonc/mdy099 PMC5961353 29635390

[94] Castroviejo-Bermejo M Cruz C Llop-Guevara A Gutiérrez-Enríquez S Ducy M Ibrahim YH. A RAD51 assay feasible in routine tumor samples calls PARP inhibitor response beyond BRCA mutation. EMBO Mol Med. (2018) 10(12):e9172. doi: 10.15252/emmm.201809172 30377213 PMC6284440

[95] Pellegrino B Herencia-Ropero A Llop-Guevara A Pedretti F Moles-Fernández A Viaplana C. Preclinical *in vivo* validation of the RAD51 test for identification of homologous recombination-deficient tumors and patient stratification. Cancer Res. (2022) 82:1646–57. doi: 10.1158/0008-5472.CAN-21-2409 PMC7612637 35425960

[96] Llop-Guevara A Loibl S Villacampa G Vladimirova V Schneeweiss A Karn T. Association of RAD51 with homologous recombination deficiency (HRD) and clinical outcomes in untreated triple-negative breast cancer (TNBC): analysis of the GeparSixto randomized clinical trial. Ann Oncol. (2021) 32:1590–6. doi: 10.1016/j.annonc.2021.09.003 34520831

[97] Blanc-Durand F Yaniz-Galende E Llop-Guevara A Genestie C Serra V Herencia-Ropero A. A RAD51 functional assay as a candidate test for homologous recombination deficiency in ovarian cancer. Gynecol Oncol. (2023) 171:106–13. doi: 10.1016/j.ygyno.2023.01.026 36868112

[98] Pikkusaari S Tumiati M Virtanen A Oikkonen J Li Y Perez-Villatoro F. Functional homologous recombination assay on FFPE specimens of advanced high-grade serous ovarian cancer predicts clinical outcomes. Clin Cancer Res. (2023) 29:3110–23. doi: 10.1158/1078-0432.CCR-22-3156 PMC10425726 36805632

[99] Compadre AJ van Biljon LN Valentine MC Llop-Guevara A Graham E Fashemi B. RAD51 foci as a biomarker predictive of platinum chemotherapy response in ovarian cancer. Clin Cancer Res. (2023) 29:2466–79. doi: 10.1158/1078-0432.CCR-22-3335 PMC10320470 37097615

[100] Kramer CJ Llop-Guevara A Yaniz-Galende E Pellegrino B Ter Haar NT Herencia-Ropero A. RAD51 as a biomarker for homologous recombination deficiency in high-grade serous ovarian carcinoma: robustness and interobserver variability of the RAD51 test. J Pathol Clin Res. (2023) 9:442–8. doi: 10.1002/cjp2.336 PMC10556259 37504067

[101] Hill SJ Decker B Roberts EA Horowitz NS Muto MG Worley MJ. Prediction of DNA repair inhibitor response in short-term patient-derived ovarian cancer organoids. Cancer Discovery. (2018) 8:1404–21. doi: 10.1158/2159-8290.CD-18-0474 PMC6365285 30213835

[102] Waks AG Cohen O Kochupurakkal B Kim D Dunn CE Buendia Buendia J. Reversion and non-reversion mechanisms of resistance to PARP inhibitor or platinum chemotherapy in BRCA1/2-mutant metastatic breast cancer. Ann Oncol. (2020) 31:590–8. doi: 10.1016/j.annonc.2020.02.008 PMC7946408 32245699

[103] Ray Chaudhuri A Callen E Ding X Gogola E Duarte AA Lee J-E. Replication fork stability confers chemoresistance in BRCA-deficient cells. Nature. (2016) 535:382–7. doi: 10.1038/nature18325 PMC4959813 27443740

[104] Franzese E Centonze S Diana A Carlino F Guerrera LP Di Napoli M. PARP inhibitors in ovarian cancer. Cancer Treat Rev. (2019) 73:1–9. doi: 10.1016/j.ctrv.2018.12.002 30543930

[105] Schlacher K Wu H Jasin M. A distinct replication fork protection pathway connects Fanconi anemia tumor suppressors to RAD51-BRCA1/2. Cancer Cell. (2012) 22:106–16. doi: 10.1016/j.ccr.2012.05.015 PMC3954744 22789542

[106] Tye S Ronson GE Morris JR. A fork in the road: Where homologous recombination and stalled replication fork protection part ways. Semin Cell Dev Biol. (2021) 113:14–26. doi: 10.1016/j.semcdb.2020.07.004 32653304 PMC8082280

[107] Berti M Cortez D Lopes M. The plasticity of DNA replication forks in response to clinically relevant genotoxic stress. Nat Rev Mol Cell Biol. (2020) 21:633–51. doi: 10.1038/s41580-020-0257-5 32612242

[108] Fuh K Mullen M Blachut B Stover E Konstantinopoulos P Liu J. Homologous recombination deficiency real-time clinical assays, ready or not? Gynecol Oncol. (2020) 159:877–86. doi: 10.1016/j.ygyno.2020.08.035 32967790

[109] Quinet A Carvajal-Maldonado D Lemacon D Vindigni A. DNA fiber analysis: mind the gap! Methods Enzymol. (2017) 591:55–82. doi: 10.1016/bs.mie.2017.03.019 28645379

[110] Maya-Mendoza A Moudry P Merchut-Maya JM Lee M Strauss R Bartek J. High speed of fork progression induces DNA replication stress and genomic instability. Nature. (2018) 559:279–84. doi: 10.1038/s41586-018-0261-5 29950726

[111] Moore KN du Bois A. Homologous recombination deficiency testing in first-line ovarian cancer. Ann Oncol. (2022) 33:231–3. doi: 10.1016/j.annonc.2021.12.013 35017033

[112] Wu M Zhu C Yang J Cheng S Yang X Gu S. Exploring prognostic indicators in the pathological images of ovarian cancer based on a deep survival network. Front Genet. (2023) 13:1069673. doi: 10.3389/fgene.2022.1069673 36685892 PMC9846244

[113] Erickson BJ. Basic artificial intelligence techniques: machine learning and deep learning. Radiologic Clinics North America. (2021) 59:933–40. doi: 10.1016/j.rcl.2021.06.004 34689878

[114] Swanson K Wu E Zhang A Alizadeh AA Zou J. From patterns to patients: Advances in clinical machine learning for cancer diagnosis, prognosis, and treatment. Cell. (2023) 186:1772–91. doi: 10.1016/j.cell.2023.01.035 36905928

[115] Shmatko A Ghaffari Laleh N Gerstung M Kather JN. Artificial intelligence in histopathology: enhancing cancer research and clinical oncology. Nat Cancer. (2022) 3:1026–38. doi: 10.1038/s43018-022-00436-4 36138135

[116] Ledesma D Symes S Richards S. Advancements within modern machine learning methodology: impacts and prospects in biomarker discovery. Curr Medicinal Chem. (2021). 28:6512–31. doi: 10.2174/0929867328666210208111821 33557728

[117] Lazard T Bataillon G Naylor P Popova T Bidard F-C Stoppa-Lyonnet D. Deep learning identifies morphological patterns of homologous recombination deficiency in luminal breast cancers from whole slide images. Cell Rep Med. (2022) 3:100872. doi: 10.1016/j.xcrm.2022.100872 36516847 PMC9798078

[118] Loeffler CML El Nahhas OSM Muti HS Seibel T Cifci D van Treeck M. Direct prediction of Homologous Recombination Deficiency from routine histology in ten different tumor types with attention-based Multiple Instance Learning: a development and validation study. medRxiv. (2023) 10:2023.03.08.23286975. doi: 10.1101/2023.03.08.23286975

[119] Bradley AP. The use of the area under the ROC curve in the evaluation of machine learning algorithms. Pattern Recognit. (1997) 30:1145–59. doi: 10.1016/S0031-3203(96)00142-2

[120] Albitar M Zhang H Pecora A Waintraub S Graham D Hellmann M. Homologous recombination abnormalities associated with BRCA1/2 mutations as predicted by machine learning of targeted next-generation sequencing data. Breast Cancer (Auckl). (2023) 17:11782234231198980. doi: 10.1177/11782234231198979 PMC10542224 37789896

[121] Zhang Y Yan C Yang Z Zhou M Sun J. Multi-omics deep-learning prediction of homologous recombination deficiency-like phenotype improved risk stratification and guided therapeutic decisions in gynecological cancers. IEEE J BioMed Health Inform. (2023). doi: 10.1109/JBHI.2023.3308440 37616142

[122] Beggs R Yang ES. Targeting DNA repair in precision medicine. Adv Protein Chem Struct Biol. (2019) 115:135–55. doi: 10.1016/bs.apcsb.2018.10.005 30798930

[123] Krais JJ Johnson N. BRCA1 mutations in cancer: coordinating deficiencies in homologous recombination with tumorigenesis. Cancer Res. (2020) 80:4601–9. doi: 10.1158/0008-5472.CAN-20-1830 PMC7641968 32747362

[124] de Almeida LC Calil FA MaChado-Neto JA Costa-Lotufo LV. DNA damaging agents and DNA repair: From carcinogenesis to cancer therapy. Cancer Genet. (2021) 252–253:6–24. doi: 10.1016/j.cancergen.2020.12.002 33340831

[125] Valieris R Amaro L Osório CAB de T Bueno AP Rosales Mitrowsky RA Carraro DM. Deep learning predicts underlying features on pathology images with therapeutic relevance for breast and gastric cancer. Cancers (Basel). (2020) 12:3687. doi: 10.3390/cancers12123687 33316873 PMC7763049

[126] Diao JA Wang JK Chui WF Mountain V Gullapally SC Srinivasan R. Human-interpretable image features derived from densely mapped cancer pathology slides predict diverse molecular phenotypes. Nat Commun. (2021) 12:1613. doi: 10.1038/s41467-021-21896-9 33712588 PMC7955068

[127] Ganzinelli M Guffanti F Ianza A Sobhani N Crovella S Zanconati F. Epithelioid Mesothelioma Patients with Very Long Survival Display Defects in DNA Repair. Cancers (Basel). (2023) 15:4309. doi: 10.3390/cancers15174309 PMC10486625 37686585

[128] Noordermeer SM van Attikum H. PARP inhibitor resistance: A tug-of-war in BRCA-mutated cells. Trends Cell Biol. (2019) 29:820–34. doi: 10.1016/j.tcb.2019.07.008 31421928

[129] Bateman NW Abulez T Soltis AR McPherson A Choi S Garsed DW. Proteogenomic analysis of enriched HGSOC tumor epithelium identifies prognostic signatures and therapeutic vulnerabilities. NPJ Precis Oncol. (2024) 8:68. doi: 10.1038/s41698-024-00519-8 38480868 PMC10937683

[130] Ghose A McCann L Makker S Mukherjee U Gullapalli SVN Erekkath J. Diagnostic biomarkers in ovarian cancer: advances beyond CA125 and HE4. Ther Adv Med Oncol. (2024) 16:17588359241233224. doi: 10.1177/17588359241233225 PMC10908239 38435431

